# Antisense sequencing improves the accuracy and precision of A-to-I editing measurements using the peak height ratio method

**DOI:** 10.1186/1756-0500-5-63

**Published:** 2012-01-24

**Authors:** Frank D Rinkevich, Peter A Schweitzer, Jeffrey G Scott

**Affiliations:** 1 Department of Entomology, Comstock Hall, Cornell University, Ithaca, NY 14853, USA; 2 Institute for Biotechnology and Life Science Technologies, Biotechnology Hall, Cornell University, Ithaca, NY 14853, USA

## Abstract

**Background:**

A-to-I RNA editing is found in all phyla of animals and contributes to transcript diversity that may have profound impacts on behavior and physiology. Many transcripts of genes involved in axonal conductance, synaptic transmission and modulation are the targets of A-to-I RNA editing. There are a number of methods to measure the extent of A-to-I RNA editing, but they are generally costly and time consuming. One way to determine the frequency of A-to-I RNA editing is the peak height ratio method, which compares the size of peaks on electropherograms that represent unedited and edited sites.

**Findings:**

Sequencing of 4 editing sites of the *Dα6 *nicotinic acetylcholine receptor subunit with an antisense primer (which uses T/C peaks to measure unedited and edited sites, respectively) showed very accurate and precise measurements of A-to-I RNA editing. The accuracy and precision were excellent for all editing sites, including those edited with high or low frequencies. The frequency of A-to-I RNA editing was comparable to the editing frequency as measured by clone counting from the same sample. Sequencing these same sites with the sense primer (which uses A/G peaks) yielded inaccurate and imprecise measurements.

**Conclusions:**

We have validated and improved the accuracy and precision of the peak height ratio method to measure the frequency of A-to-I RNA editing, and shown that results are primer specific. Thus, the correct sequencing primer must be utilized for the most dependable data. When compared to other methods used to measure the frequency of A-to-I RNA editing, the major benefits of the peak height ratio are that this method is inexpensive, fast, non-labor intensive and easily adaptable to many laboratory and field settings.

## Findings

A-to-I RNA editing is catalyzed by adenosine deaminases that act on RNA (ADARs) that bind to double stranded pre-mRNAs and convert adenosine (A) to inosine (I) which is recognized by the ribosome as a guanosine [1]. ADARs are found in all animals, but are absent from protists, plants and fungi [2]. There are three *ADAR *genes in vertebrates. *ADAR1 *and *ADAR2 *both have catalytic activity, whereas *ADAR3 *lacks activity, although the functional domains of *ADAR3 *are conserved. *ADAR3 *is likely is a duplicate of *ADAR2*. A single *ADAR *gene exists in insects. *Drosophila melanogaster dAdar *is homologous to vertebrate *ADAR2 *[2].

A-to-I RNA editing regulates behavior and life history traits in many phyla of animals. The widespread conservation of this pathway is thought to be a viral defense mechanism [3,4]. A-to-I RNA editing occurs in protein coding and non-coding sequences, transposable elements, introns, 5' and 3' untranslated regions of the pre-mRNA that may result in changes in the amino acid sequences, splice sites or levels of transcripts [4,5]. RNA editing frequently results in non-synonymous substitutions that can be critically important for proper function or tissue distribution. For example, RNA editing results in a Q/R amino acid substitution in the pore loop domain of the AMPA receptor GluR-B subunit that makes the channel impermeable to Ca^2+^. Unedited GluR-B transcripts lead to neuronal death that causes seizures and premature death in editing deficient mice [6].

The transcripts of many ligand-gated or voltage-sensitive ion channels and G-protein coupled receptors are targets of A-to-I RNA editing [4,7-9]. Genome wide studies in *D. melanogaster *have shown wide-spread editing of these genes [9]. RNA editing of these genes is most common in regions that code for functionally important amino acids in the protein. In voltage-gated K^+^, Na^+ ^and Ca^2+ ^channels, residues involved in channel gating or inactivation are edited. Editing sites on nAChR or GABA receptor subunits occur in crucial areas in the ligand-binding domain and TM2 that forms the channel pore [9-12].

There are a number of *dAdar *transcripts produced and *dAdar *expression is important for a number of functions in the fruit fly. Four common transcripts of *dAdar *result from alternative splicing and these transcripts are themselves subject to RNA editing [13]. *D. melanogaster dAdar *null mutants show significant deficiencies in motor control and mating that grow progressively worse with age. Nervous system morphology is greatly affected by *dAdar *null mutants [14]. Behavioral deficits were also seen in *D. melanogaster *that had reduced *dAdar *expression. These mutants did not fly, exhibited diurnal activity patterns and displayed temperature sensitive paralysis [15]. Males deficient in *dAdar *took longer to engage in courtship behaviors and had a dramatically altered courtship song [16].

There are a variety of methods for measuring the extent of A-to-I RNA editing [17,18] and these are important for the burgeoning study of A-to-I RNA editing (since 2007, there have been more than 1080 journal articles, book chapters and meeting presentations on A-to-I RNA editing). However, current methods to measure the extent of RNA editing tend to be costly, time consuming and use radioactive materials or generate hazardous waste. Using the *Dα6 *nicotinic acetylcholine receptor subunit, we demonstrate a detailed and improved method for a highly accurate and precise estimate of the frequency of editing using peak height ratios from Sanger sequencing electropherograms and compare its costs and benefits to other methods for measuring RNA editing frequency.

## Methods summary

We used the peak height ratio method to estimate the level of A-to-I RNA editing at 4 sites of the *Dα6 *nicotinic acetylcholine receptor subunit cDNA from the Canton-S strain of *Drosophila melanogaster*. *Dα6 *clones that were unedited or edited at four sites were mixed in different proportions by weight to produce varying levels of A-to-I RNA editing. Two primers, (D*α*6IR2 and D*α*6285F, Table 1) were used for sequencing. D*α*6IR2 was an antisense primer and D*α*6285F is a sense primer. The D*α*6IR2 and D*α*6285F primers yielded a mix of T/C or A/G electropherograms peaks, respectively, to indicate unedited and edited sites. The heights of the peaks at each editing site from the electropherogram were measured using Photoshop Creative Suite 4 (Adobe Systems Inc, San Jose CA) and the ratio of the peak heights was compared to the expected heights [17]. The reliability of the peak height ratio method was validated by comparing the frequency of editing determined from sequencing individual clones from the same sample. A more detailed description of the methods used is found in the Additional File 1.

**Table 1:** Sequences of the primers used

Primer Name	Sequence
Dα6ORF-F	CACGCGATACAAACAAGCCAAGGACA
Dα6ORF-R	ACGATTATGTGCGGAGCGGAGAG
DmelActinF	ACTCCGGCGATGGTGTCTCC
DmelActinR	GGGCGGTGATCTCCTTCTGC
T7	TAATACGACTCACTATAGGG
SP6	TATTTAGGTGACACTATAG
Dα6R	CCAGGGCAGCCATTGTAGGAAAAC
Dα6IR2	GCAGCAGGCGTAGACTATCGTATT
Dα6285F	AACGGAATACGGCGGGGTCAAG
Dα6ORF-F3	GCGCCTGCTGAACCATCTGC
Dα6ORF-R3	ACCACCGACGAGGGCGACCAT

### Determination of editing with Dα6IR2

**Figure 1:**
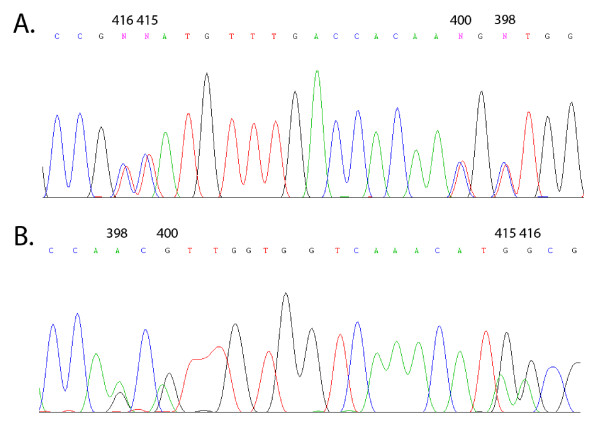
**Electropherograms of a sample containing a 1:1 mixture of clones that are edited and unedited**. Electropherograms representing 1:1 ratios of edited and unedited transcripts sequenced with **A**) Dα6IR2 (antisense) or **B**) Dα6285F (sense) primers. Numbers above peaks indicate editing sites based on the nucleotide numbering of the open reading frame of Dα6. Note that Dα6IR2 is an antisense primer so the sequence of A-to-I editing sites is in reverse. Antisense sequencing with Dα6IR2 generates editing sites as a mix of C/T signals, whereas sense sequencing with Dα6285F generates editing sites as a mix of A/G signals.

Determination of *Dα6 *editing at four sites was very accurate and precise when sequenced with Dα6IR2 (Figure 1A), and observed values never varied from the expected values by more than 3% (Table 2). The slope of the line from a plot of observed vs. expected editing (for each site) was not significantly different from 1.0 and the r^2 ^values were 1.0 (Table 3). Therefore, the observed frequency of editing is in high agreement with the expected frequency of editing at all sites.

**Table 2:** Estimates of A-to-I RNA editing frequency between Dα6IR2 and Dα6285F sequencing primers

		Editing Site			
		
Sequencing Primer	Expected Editing Rate	398	400	415	416
Dα6IR2	0.95	0.94 ± 0.02	0.96 ± 0.01	0.92 ± 0.03	0.92 ± 0.03
	0.90	0.88 ± 0.01*	0.90 ± 0.01	0.87 ± 0.00*	0.88 ± 0.01
	0.75	0.76 ± 0.01	0.74 ± 0.01	0.75 ± 0.02	0.76 ± 0.01
	0.50	0.51 ± 0.01*	0.50 ± 0.03	0.50 ± 0.02	0.52 ± 0.01*
	0.25	0.25 ± 0.03	0.24 ± 0.02	0.23 ± 0.01	0.25 ± 0.02
	0.10	0.11 ± 0.03	0.09 ± 0.01	0.09 ± 0.02	0.09 ± 0.03
	0.05	0.07 ± 0.03	0.07 ± 0.02	0.08 ± 0.05	0.07 ± 0.05
Dα6285F	0.95	0.85 ± 0.12	0.88 ± 0.17	0.97 ± 0.03	0.97 ± 0.02
	0.90	0.86 ± 0.05	0.86 ± 0.05	0.95 ± 0.01*	0.93 ± 0.00*
	0.75	0.66 ± 0.04*	0.79 ± 0.04	0.84 ± 0.03*	0.81 ± 0.03*
	0.50	0.38 ± 0.02*	0.58 ± 0.02*	0.67 ± 0.01*	0.60 ± 0.05*
	0.25	0.19 ± 0.03*	0.33 ± 0.04*	0.43 ± 0.04*	0.39 ± 0.13
	0.10	0.06 ± 0.03	0.17 ± 0.02*	0.33 ± 0.06*	0.23 ± 0.10*
	0.05	0.04 ± 0.02	0.13 ± 0.04	0.25 ± 0.04*	0.23 ± 0.06*

Values represent the mean ± standard deviation. Values with an asterisk indicate estimated editing frequency is different from the expected editing rate (one-sample *t*-test vs. expected mean, *p *< 0.05)

**Table 3:** Comparison of the reliability of estimating A-to-I RNA editing of the Dα6 subunit using the peak height ratio method between Dα6IR2 and Dα6285F primers at different rates of expected editing

	Editing Site			
	
Seq. Primer	398	400	415	416
Dα6IR2	0.98 ± 0.03 (1.00)	1.00 ± 0.03 (1.00)	0.97 ± 0.05 (1.00)	0.97 ± 0.05 (1.00)
Dα6285F	0.94 ± 0.10 (0.99)	0.88 ± 0.10* (0.99)	0.80 ± 0.07* (0.99)	0.85 ± 0.04* (1.00)

Values are the slopes and associated confidence intervals of editing estimate vs. expected values. Numbers in parentheses indicate the r^2 ^value. Slopes with * indicate values that are significantly different than 1.0 (i.e., 95% CI does not include 1.0)

### Determination of editing with Dα6285F

Determination of *Dα6 *editing estimates was unreliable (inaccurate and imprecise) when sequenced with Dα6285F at all four editing sites (Figure 1B, Table 2). The observed frequency of editing was significantly different than expected in 17 of 28 comparisons and varied by as much as 23% (Table 2). In all, 20 estimates were significantly different from the expected editing rate by more than 5%. The slope of the line from a plot of observed values of editing vs. those expected was significantly different from 1.0 for three of the four editing sites and the r^2 ^value was less than 1.0 in 3 of the 4 cases (Table 3). This indicates use of the sense Dα6285F primer is not a reliable method for determining the frequency of editing.

### Validation of the peak height ratio method using a known sample

**Figure 2:**
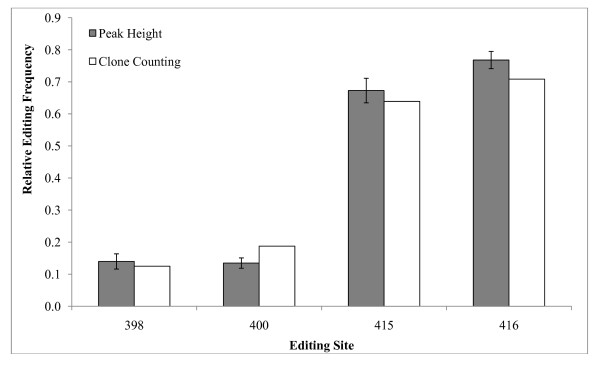
**Comparison of peak height ratio and clone counting methods**. Comparison of editing estimates between the peak height ratio method and clone counting at four editing sites of *D*α*6*. There is no difference in the editing proportion of samples using the peak height ratio method or clone counting. The editing site numbers represent the base of the open reading frame of *D*α*6 *that is edited.

The frequency of editing at 4 sites was determined for a sample of Canton-S cDNA by examining the sequences of individual clones determined with the Dα6IR2 primer. This sample was then evaluated using the peak height ratio method. Comparison of these methods showed they were in close agreement and were not significantly different at any editing site. This agreement was observed for sites that had either a low (398 and 400) or high frequency of editing (415 and 416, Figure 2).

## Method comparison

We have validated and verified a very accurate, precise, fast and cost-effective method for estimating editing rates. The accuracy and precision of the editing estimates were primer specific (Figures 1 and 2, Tables 2 and 3). Results with primer Dα6IR2 were excellent, while those with primer D*α*6285F were much less reliable (Figure 1). When sequenced with D*α*6IR2, unedited and edited sites appeared as a mix of T/C signals, respectively. In contrast, results with D*α*6285F showed unedited and edited sites as A or G, respectively (Figure 1B, Table 2). Therefore, it is imperative that the proper primer (D*α*6IR2 in this case) be used to accurately estimate the frequency of A-to-I RNA editing.

The disparity in editing estimation between primers may be a consequence of the sequencing chemistry commonly used for dye-terminator cycle sequencing prior to analysis on automated, capillary DNA analyzers. Current nucleotide formulations substitute dITP for dGTP to prevent compression of poly-G tracts in the resulting electropherogram. However, the incorporation of dITP is less efficient than dGTP [19].

Table 4 shows a comparison of the time, labor and financial costs of the peak height ratio method with existing protocols. The financial cost savings are the most significant and noteworthy advantage of the peak height ratio method. Other major benefits (which can result in additional savings) of the peak height ratio method are that nearby editing sites can be evaluated simultaneously (Table 4), requires only 6 steps to generate data from a sample (every other method requires more steps, all of which can introduce data variation and experimental failure), is relatively fast, and can be carried out with equipment normally available in most labs (only a centrifuge and a thermocycler are needed).

**Table 4:** Comparison of methods used to measure A-to-I RNA editing

Method	# Steps	Days	Cost/Sample	Cost/ 3 Analyses^*a*^	Relative Cost	Sites/ Analysis
Peak Height Ratio	6 (RNA isolation, RT, PCR, PCR purification, sequencing, analysis)	3	$11.87	$35.62	1	Several

Poisoned Primer Extension	7 (RNA isolation, RT, primer labeling, PCR, gel, imaging, analysis)	1-2	$120.63 F^*b *^$123.40 ^32^P	$133.24 F^*b *^$157.84 ^32^P	15.0 F^*b*^ 17.7 ^32^P	One

Restriction Digest	9 (RNA isolation, RT, PCR, purification, RE digestion, purification, gel, imaging, analysis)	2	$10.37 F^*b*^ $123.40 ^32^P	$31.10 F^*b*^ $135.70 ^32^P	3.50 F^*b*^ 15.2 ^32^P	One

Ultra High Throughput Sequencing	9 (RNA isolation, RT, PCR(×2), purification(×2), hybridization, sequencing, analysis)	21-28	$1609.80	$4829.39	136	One to Several^*c*^

Clone Counting	10 (RNA isolation, RT, PCR, PCR purification, cloning, transformation, colony screening, colony growth, plasmid purification, sequencing, analysis)	6	$24.48	$561.25	15.8	Several

*^a ^*= Cost/3 Analyses is simply not three-fold the Cost/Rep as primers can be labeled and used in multiple samples on a single day of experiments

*^b ^*= Fluorescent assay

*^c ^*= the ability to read several editing sites is determined by proximity of editing sites

Costs are based on using the materials listed in Additional File 1. Additional costs were based on pGEM-T Cloning System II, PureYield Plasmid Purification System (Promega), restriction enzymes (PshAI and HpyCH4V, New England Biolabs), KinaseMax Kit, Alexa Fluor 488 and NucAway (Invitrogen). Data for Ultra High Throughput Sequencing is based on Abbas et al., 2010 and consists of a single lane 86 bp single-end read on a Genome Analyzer. The cost/sample is the cost of performing a single experiment from one biological sample. The cost/3 analyses is the cost for three replicates from one biological sample. Relative cost is for assessing the four editing sites of *Dα6 *reported herein. The price of shipping, primers, gels, standard markers, imaging equipment, software, and labor were not included in the cost

Besides these savings of time and money, there are many other technical advantages of using peak height ratios over other methods. RNA editing may introduce or eliminate a restriction enzyme site. Poisoned primer extension utilizes ddGTP as a reaction terminator in edited transcripts. A larger band will result from unedited transcripts because dATP would be incorporated into the product. These methods utilize fluorescent or phosphoimaging systems for quantification.

The major drawbacks to poisoned primer extension and restriction digests are that they only allow for the quantification of a single editing site, they use radiolabeled or fluorescently labeled primers, and are somewhat labor intensive. The high cost of the restriction digest with radioisotopes and poisoned primer extension is mainly due to the high cost of fluorescent dyes and ^32^P. These two protocols are cost effective only when larger numbers of replications can be performed in a short period of time. In the case of restriction enzyme digestion, the edited site may alter a restriction enzyme recognition sequence for which an inexpensive or widely used restriction enzyme may not be readily available. In the case of adjacent or nearby editing sites, multiple enzymes would be needed to be used to account for recognition site variation. Complications may arise in cases when a restriction enzyme is not available to recognize the change in recognition sequence.

A major limitation of the poisoned primer extension method is that is can only assess one editing site at a time. In order to obtain the same amount of data across four editing sites as used in this experiment, four unique reactions would need to be run on each sample. This would require extensive sample planning and management. Also, using sense primers to assess editing at adjacent editing sites would be particularly troublesome, as in the case of editing sites 415 and 416 of the *Dα6 *nAChR subunit used in this experiment. Two sense primers would have to be utilized to account for transcripts with edited and unedited versions at site 415 in order to accurately assess editing at site 416 as mismatches at the 3' end of primer with template can lead to reduced amplification efficiency. Conversely, an antisense primer could be used to assess editing at site 416 without regard for editing status at site 415. However, this would require the use of ddCTP as the reaction terminator. The cost to measure the same 4 editing sites of *Dα6 *by poisoned primer extension would be 15 to 17 fold higher than by the peak height method (Table 4).

Ultra high throughput sequencing (UHTS) is extremely cost prohibitive and better suited for experiments that may not require comparing many biological samples. Next generation UHTS is the most accurate method to measure the frequency of RNA editing. It can even detect rare transcripts that are missed by clone counting methods [18]. The major disadvantages are cost and the short reads generated may only be useful for multiple editing sites that are nearby if the user needs to know what sites are edited on a specific transcript. However, the undeniable major advantage of UHTS is that many editing sites on many transcripts of many genes can be evaluated.

Clone counting can be performed to quantify the frequency of editing. While there are many advantages to this method, the major drawback is that a large number of clones need to be sequenced to ensure an accurate reflection of editing frequency. This process may take a few days to complete and the cost of sequencing a large number of clones may be substantial. Screening colonies for positive inserts usually requires screening many more colonies than will actually go for sequencing. The waste generated by growing colonies on plates and in liquid media needs to properly disposal by autoclaving or incinerating at an approved facility.

The peak height ratio method with the antisense primer utilizes the different intensities in the T/C signal of eletropherograms. The disadvantage of this method is that it does not allow for the identification of which editing sites are edited on each transcript. However, the cost, labor, turnaround time is unequivocal. The peak height ratio method is very advantageous in that there are few steps required to complete an analysis from biological sample to data point.

## Applications of this method

The technical efficiency of the peak height ratio method makes it an ideal method for demonstrating the extent of A-to-I RNA editing in high school and introductory biology classes, or in research laboratories as a significant cost reduction method. Additionally, a mobile lab unit could be assembled for rapid sample processing in the field and other remote areas. The RNA extraction, reverse transcription and PCR could be done in the field and processed samples could be mailed to a sequencing facility. Processing field collected animals would help overcome potential changes in allele frequency that may result from genetic drift if they were returned to a lab and reared for a second generation. This would also reduce the risk of sample degradation and shipping nucleic acids across international borders is typically less burdensome than shipping organisms or tissues.

The peak height method could also be used to estimate allele frequencies within populations. Using pools of animals, it is possible to simultaneously evaluate allele frequencies from as many as 10 diploid or 20 haploid individuals based on the upper (0.95) and lower (0.05) detection rates that we used in this analysis. This would allow for the detection of a single allele out of 20 potential alleles. It is likely that this method could be validated for even lower detection rates. This method would be extremely valuable in our lab for evaluating the frequency of insecticide resistance alleles from field collected populations.

## Conclusions

The accuracy and precision of the estimate of A-to-I RNA editing using the peak height ratio method with sequences of *Dα6 *from the antisense D*α*6IR2 primer is in very good agreement with expected values and is comparable to the quantitative clone counting method. It is also very cost effective and fast compared to other current methods, especially when evaluating editing at multiple sites. Because of these many advantages, it is likely that this method will prove to be a powerful and useful tool in evaluating the extent of A-to-I RNA editing in this rapidly growing field of study, and has other uses in other fields (such as population genetics) as well.

## Competing interests

The authors declare that they have no competing interests.

## Authors' contributions

FDR and JGS designed the experiments and wrote the manuscript. FDR performed all laboratory work and data analysis. PAS provided technical commentary and analysis of sequencing methodology. All authors approved of the final manuscript.

## Supplementary Material

Additional file 1 **Detailed Methods**. Click here for file
