# Supplementary material for: Antisense sequencing improves the accuracy and precision of A-to-I editing measurements using the peak height ratio method
Source: BMC Res Notes. 2012 Jan 24;5:63. doi: 10.1186/1756-0500-5-63 (PMC3296654; doi:10.1186/1756-0500-5-63)
Supplement: Additional file 1 — Detailed Methods. [file 1756-0500-5-63-S1.PDF]

MATERIALS AND METHODS

RNA isolation, Dα6 PCR, Cloning and Sequencing

Total RNA was isolated from 3 batches of 10, 1-3 day old Canton-S flies (5♀ and 5♂) using TRIzol (Invitrogen, Carlsbad CA) according to the manufacturer's directions. The RNA was dissolved in 50 ul of DEPC-treated water. Reverse transcription was performed with 5 ug of total RNA using Go-Script (Promega, Madison WI) according to the manufacturer's directions.

A 1502 bp fragment of the open reading frame of the *Dα6* nicotinic acetylcholine receptor subunit was amplified in 100 ul Go-Taq reactions with 2 ul of cDNA and 2 ul of each primer Dα6ORF-F and Dα6ORF-R (Table 1). A control reaction for a 497 bp fragment of *actin5c* was performed under the same reaction conditions with the primers DmelActinF and DmelActinR (Table 1). Thermocycler conditions were as follows: 95°C for 2 min, 40 cycles of 95°C for 30 s, 56°C for 30 s, and 72°C for 1.5 min, with a final extension at 72°C for 10 min. PCR products were visualized on a 1% agarose gel stained with 50 ug/ml of ethidium bromide under UV light. PCR products were purified using the Wizard PCR Purification Kit (Promega, Madison WI). Up to 100 ng of the purified *Dα6* open reading frame cDNA were cloned into pGEM-T vector (Promega, Madison WI) according to the manufacturer's directions, except that ligation was carried out at 4°C overnight. JM109 competent cells were transformed via heat shock with two ul of the cloning reaction and spread on Luria Beranti plates containing ampicillin (100 ug/ml), X-Gal (40 ug/ml) and ITPG (100 uM) and grown at 37°C overnight. White colonies were screened for the presence of the correct insert by spotting the colony to a fresh plate with a 10 ul pipette tip and using the pipette tip directly in a 15 ul Go-Taq reaction with T7 and SP6 primers (Table 1). The conditions for colony PCR were as follows: 95°C for 2 min, 35 cycles of 95°C for 30 s, 42°C for 30 s, and 72°C for 2 min, with a final extension at 72°C

for 10 min. PCR products were visualized on a 1% agarose gel stained with 50 ug/ml of ethidium bromide under UV light. Positive colonies were incubated at 37°C overnight in 3 ml of Luria broth medium containing 100 ug/ml ampicillin. Plasmid DNA was isolated using the Wizard Plus SV Minipreps (Promega, Madison WI) and eluted in 100 ul H2O. Positive clones were sequenced from both ends using T7 and SP6 primers as well as Dα6R (Table 1) at Cornell's Biotechnology Resource Center on an Applied Biosystems Automated 3730 DNA Analyzer using Big Dye Terminator chemistry and AmpliTaq-FS DNA Polymerase. Sequences for each clone were aligned using MegAlign (DNA Star, Madison, WI).

Validation of Peak Height Ratio Method Using Clones

Dα6 clones that were entirely edited or unedited at editing sites 398, 400, 415, and 416 (based on the nucleotide numbering of the open reading frame of *Dα6*) were identified [19]. We did not observe editing at site 413 in any samples (n=17) in these experiments. Different amounts (by weight) of plasmid DNA from clones that were completely edited or unedited were added to sequencing tubes to provide known ratios of edited and unedited sites to assess 5, 10, 25, 50, 75 90, and 95% editing rates. Plasmids were sequenced with the primers Dα6IR2 (antisense) and Dα6285F (sense, Table 1). The heights of the peaks at each editing site were measured using Photoshop Creative Suite 4 (Adobe Systems Inc, San Jose CA) and the ratio of the peak heights was compared to the expected heights (based on the ratio of the clones used). The accuracy of the estimates was statistically compared with the expected value with a 1-sample t-test against the expected value. The slope of the expected versus estimated editing rate was calculated using linear regression in Excel.

Validation of Peak Height Ratio Method Using a Known Sample

A fragment of *Dα6* was amplified from three separate cDNA samples from Canton-S with the primers Dα6ORF-F3 and Dα6ORF-R3 in 100 ul Go-Taq reactions under the following thermocycler conditions: 95°C for 2 min, 35 cycles of 95°C for 30 s, 55°C for 30 s, and 72°C for 30 s, with a final extension at 72°C for 10 min. PCR products were purified and sent for sequencing with the Dα6IR2 primer as described above. Editing estimates were calculated based on peak height ratios as described above. The results from this method were compared to the rate quantified from individual clones isolated as described above.
